# The non-oral infection of larval *Echinococcus granulosus* induces immune and metabolic reprogramming in the colon of mice

**DOI:** 10.3389/fimmu.2022.1084203

**Published:** 2023-01-13

**Authors:** Yuying Zhou, Tiancheng Luo, Yuying Gong, Yuxin Guo, Dingmin Wang, Zixuan Gao, Fenfen Sun, Linlin Fu, Hua Liu, Wei Pan, Xiaoying Yang

**Affiliations:** ^1^ Jiangsu Key Laboratory of Immunity and Metabolism, Department of Pathogen Biology and Immunology, Jiangsu International Laboratory of Immunity and Metabolism, Xuzhou Medical University, Xuzhou, Jiangsu, China; ^2^ The First Clinical Medical College, Xuzhou Medical University, Xuzhou, Jiangsu, China; ^3^ The Second Clinical Medical College, Xuzhou Medical University, Xuzhou, Jiangsu, China; ^4^ Department of Histology and Embryology, Basic Medical College, Xuzhou Medical University, Xuzhou, Jiangsu, China; ^5^ National Institute of Parasitic Diseases, Chinese Center for Disease Control and Prevention, Chinese Center for Tropical Diseases Research, National Health Commission (NHC) Key Laboratory of Parasite and Vector Biology, World Health Organization (WHO) Collaborating Centre for Tropical Diseases, National Center for International Research on Tropical Diseases, Shanghai, China

**Keywords:** larval *E. granulosus*, colon, transcriptomics, immune response, metabolic reprogramming

## Abstract

**Background:**

The intestinal tract serves as a critical regulator for nutrient absorption and overall health. However, its involvement in anti-parasitic infection and immunity has been largely neglected, especially when a parasite is not transmitted orally. The present study investigated the colonic histopathology and functional reprogramming in mice with intraperitoneal infection of the larval *Echinococcus granulosus* (*E. granulosus*).

**Results:**

Compared with the control group, the *E. granulosus*–infected mice exhibited deteriorated secreted mucus, shortened length, decreased expression of tight junction proteins zonula occludens-1 (ZO-1), and occludin in the colon. Moreover, RNA sequencing was employed to characterize colonic gene expression after infection. In total, 3,019 differentially expressed genes (1,346 upregulated and 1,673 downregulated genes) were identified in the colon of infected mice. KEGG pathway and GO enrichment analysis revealed that differentially expressed genes involved in intestinal immune responses, infectious disease-associated pathways, metabolism, or focal adhesion were significantly enriched. Among these, 18 tight junction-relative genes, 44 immune response-associated genes, and 23 metabolic genes were annotated. Furthermore, mebendazole treatment could reverse the colonic histopathology induced by *E. granulosus* infection.

**Conclusions:**

Intraperitoneal infection with *E. granulosus* induced the pathological changes and functional reprogramming in the colon of mice, and mebendazole administration alleviated above alternations, highlighting the significance of the colon as a protective barrier against parasitic infection. The findings provide a novel perspective on host-parasite interplay and propose intestine as a possible target for treating parasitic diseases that are not transmitted orally.

## Introduction

The intestinal tract serves as a critical regulator for nutrient absorption and overall health (1–3). Recent advances in immunometabolism reinforce the importance of intestinal homeostasis in maintaining host immune and metabolic balance, thereby highlighting the potential of intestine serves as a therapeutic target for many diseases (4, 5). To avoid the intrusion of pathogenic component, the intestine establishes a complicated physical and chemical barrier function (6, 7), including production of mucus by goblet cells, expression of tight junction proteins by intestinal epithelial cells (8), and Paneth cell–derived antimicrobial peptides (3). It is well established that gut barrier dysfunction usually initiates from intestinal component deficiency (4, 9), characterized by increased permeability and impaired defense function. Leaky gut provides a gateway for intestinal and extra-intestinal substance to crosstalk, especially luminal pathogens and antigenic molecules (7, 10). For example, hyper-translocation of bacterial lipopolysaccharide (LPS, endotoxin) into the blood circulation may trigger immune response resulting in upregulation of pro-inflammatory cytokines-related mRNA expression (7, 9). Therefore, the optimal intestinal immune response guarantees the proper immediate environment for host immunometabolism, reducing inflammation and improving intestinal barrier integrity (3, 11).

Parasites, a major kind of pathogens obtaining nutrients from the hosts, have been reported to affect host immune and metabolism in subtle ways (12–14). It is documented that *N. americanus* hookworms infection may influence mucosal immune response *via* damaging gut barrier intact and inducing parasite-specific immunosuppression (15). Since intestine is regarded as the largest immune organ in the body, intestinal-parasite relationships have grown to be a novel topic of parasitic research in recent years (16–18). Delineating host immunity against parasites and how parasites escaping immune attack is of obvious importance to human well-being (14). However, the majority of studies focus on orally infected parasites; the function of intestinal tract in non-orally transmitted parasites infection has been largely neglected.

*Echinococcus granulosus* (*E. granulosus*) is a representative helminth of medical importance as the pathogen of cystic echinococcosis (CE), widely known for its high host adaptability and worldwide distribution (19). Emerging evidence indicates that the parasite has evolved novel strategies to subvert host immune responses (12). Our previous studies revealed that *E. granulosus* infection disturbs lipid metabolism, resulting in a decrease in subcutaneous fat and reduced adipocytes size (20). Moreover, the excretory-secretory products (ESPs) derived from the *E. granulosus* could induce the differentiation of immunosuppressive cells to downregulate the immunity against parasitic infection (12, 13, 21). In previous studies, orally infected model was widely employed to investigate *E. granulosus*-associated intestinal alternations (22, 23). However, the impact of *E. granulosus* infection on intestine during non-oral transmission remains elusive.

In the present study, using a non-oral transmission model of the *E. granulosus* by intraperitoneal injection of protoscoleces (PSCs) to mice, we evaluated the effects of *E. granulosus* infection on colonic pathology and functional reprogramming. The pathological changes of colon were analyzed by colonic barriers function. The functional reprogramming of colon was examined by RNA sequencing. Meanwhile, mebendazole (MBZ), a priority drug of CE (24), was administrated to evaluate the function of intestinal tract during *E. granulosus* infection. Our finding indicates a neglected role of intestine immune and metabolic homeostasis in the pathogenesis of larval **E. granulosus*.*

## Materials and methods

### Parasite preparation

The PSCs of *E. granulosus* (EgPSC) were acquired from hydatid cysts of sheep naturally infected with *E. granulosus* as described previously (13). The non-oral transmission model of the *E. granulosus* were performed as we mentioned before (25). Briefly, the EgPSC from naturally infected sheep were washed with PBS for three times and resuspended in sterile saline supplemented with penicillin (100 μg/ml, Beyotime Biotech, Beijing, China) and streptomycin (100 U/ml, Beyotime Biotech, Beijing,China).

### Animal studies

Female C57BL/6J mice aged 6 weeks were purchased from Shanghai Laboratory Animal Center (SLAC, Shanghai, China) and maintained in the Experimental Animal Center of Xuzhou Medical University with a conditional environment (12h light/12h dark cycle, free access to water and food). The protocols of all animal care and experiments were authorized by the Laboratory Animal Welfare and Ethics Committee (LAWEC) of Xuzhou Medical University (Xuzhou, China, SCXK [Su] 2020–0048). For detecting the influence of *E. granulosus* infection on intestine, mice were randomly assigned to two groups (*n* = 6 per group) (1): mice intraperitoneally injected with 200 μl of sterile saline as the control group and (2) mice intraperitoneally injected with 200 μl of sterile saline containing 2000 living EgPSC as the infected group. For investigation of MBZ’s effect on intestine in infected mice, mice were randomly assigned to three groups (*n* = 6 per group): (1) mice intraperitoneally injected with 200 μl of sterile saline for 15 consecutive days as the control group, (2) mice intraperitoneally injected with 200 μl of sterile saline for 14 consecutive days post–*E. granulosus* infection as the infected group, and (3) mice intraperitoneally injected with 12.5 mg/kg MBZ (Sigma-Aldrich, St. Louis, MO, United States) for 14 consecutive days post–*E. granulosus* infection as the treated group. Seven months after infection, mice were anesthetized with chloral hydrate, and the colonic tissues were collected for further analysis.

### Periodic acid-Schiff staining

Periodic acid-Schiff (PAS) staining was performed according to the established protocol (26). Briefly, paraffin-embedded colonic tissues were cut into 5-μm sections and oxidized in 0.5% periodic acid for 5 min after de-paraffinization. Slides were then rinsed in distilled water, placed in Schiff reagent, counterstained in Mayer’s hematoxylin, mounted onto slides and then visualized microscopically. The amounts of PAS-positive cells (goblet cells) per colonic crypt were counted in at least 10 randomly selected PAS-stained crypts with sagittal orientation (27).

### Transcriptome profiling

Fresh colonic samples of three control and three infected mice were collected to analyze the whole profile of transcriptome. Sequencing and data analysis were conducted in CapitalBio Technology Co., Ltd. (Beijing, China). Total RNA was extracted from each sample using Trizol reagent kit (Invitrogen, Carlsbad, CA, USA) according to the manufacturer’s instruction. Then, the total mRNA was enriched by Oligo (dT) magnetic beads. The mRNA sequences were fragmented into debris by using fragmentation buffer and reverse-transcribed into first-strand cDNA, following by second-strand cDNA amplification by polymerase chain reaction (PCR). The ligation debris were size selected by agarose gel electrophoresis and amplified and sequenced by using Illumina HiSeqTM 2500 platform. DESeq2 was used to determine the gene expression differentiation between samples and obtained a *P*-value. Corrected *P*-value (*q*-value) was calculated by correcting using BH method. *P*-value or *q*-value was used to conduct significance analysis. Parameters for classifying significantly differentially expressed genes (DEGs) are ≥ two fold differences (|log_2_FC| ≥ 1, FC: the fold change of expressions) in the transcript abundance and *q* < 0.05 (28, 29).

### Gene Ontology and Kyoto Encyclopedia of Genes and Genomes enrichment analysis

The Gene Ontology (GO) analysis was performed to identify the potential biological functions based on the different expression level of mRNAs. Three parts were involved in the GO terms, including biological process (BP), cellular component (CC), and molecular function (MF). Additionally, the Kyoto Encyclopedia of Genes and Genomes (KEGG) pathway analysis was also conducted to forecast the possible signal pathways *via* analyzing the distinctively expressed genes.

### Quantitative reverse transcription polymerase chain reaction

Quantitative reverse transcription PCR (qPCR) was conducted to verify the transcriptome results. The exact procedure was described in the previous study (4). In brief, total RNA was extracted from homogenized colonic tissues in TRIzol^®^ (Vazyme Biotech Co., Ltd., Nanjing, China). One-microgram purified RNA of each sample was firstly reverse-transcripted to cDNA using a High-Capacity cDNA Reverse Transcription Kit (Vazyme Biotech Co., Ltd., Nanjing, China). qPCR was performed using LightCycler^®^ 480 II Real-time PCR Instrument (Roche, Swiss). All samples were repeated for three times, and the relative expression of related genes was normalized relative to the endogenous reference (β-actin) with the 2^-ΔΔCt^ method. Primer sequences were listed in **Table 1**.

**Table 1:** The qPCR primer sequences involved in the study.

Primer names	Sequences (5′to 3′)	
Cldn8	Forward:	GCAACCTACGCTCTTCAAATGG
	Reverse:	TTCCCAGCGGTTCTCAAACAC
Pklr	Forward:	CCCGAGATACGCACTGGAG
	Reverse:	CGACCTGGGTGATATTGTGGT
Cldn4	Forward:	GGAGGGCCTCTGGATGAACT
	Reverse:	GATGCTGATGACCATAAGGGC
Cxcl12	Forward:	TGCATCAGTGACGGTAAACCA
	Reverse:	CACAGTTTGGAGTGTTGAGGAT
β-actin	Forward:	AGAAGGTGGTGAAGCAGGCATC
	Reverse:	CGAAGGTGGAAGAGTGGGAGTTG

### Western blot analysis

Proteins were extracted from homogenized colonic tissues of mice using ice-cold RIPA lysis buffer containing complete EDTA-free protease inhibitor cocktail and PhosSTOP Phosphatase Inhibitor as previous (5). After centrifugation, the supernatants were collected, and protein concentrations were quantitated by BCA assay (Beyotime Biotech, Beijing, China). Forty-microgram protein of each sample were separated by 10% SDS-PAGE and electro-transferred onto polyvinylidene difluoride (PVDF) membranes (BioRad, Hercules, CA, United States). Membranes were blocked with 5% defatted milk and incubated with indicated primary antibodies at 4°C for 12h. Then, membranes were incubated with HRP-conjugated anti-rabbit IgG secondary antibody (#7074, CST, Boston, MA, United States) at room temperature for 1h. Finally, the protein bands were test using Clarity™ ECL Western blot substrate (1705,060, Bio-Rad, Hercules, CA, United States) and captured using the ChemiDoc Touch imaging. Primary antibodies and dilutions used were anti–β-actin (AC026, ABclonal Biotechnology Co., Ltd., Wuhan, China, 1:50000), anti-ZO-1 (ab96587, Abcam, Cambridge, United Kingdom, 1:1000), and anti-occludin (ab167161, Abcam, Cambridge, United Kingdom, 1:1000).

### Statistical analysis

All data in this study were calculated using the software Graphpad Prism 8.0 (GraphPad Software, San Diego, CA, United States) and presented as mean ± SEM. Statistical significance was performed using the unpaired tailed Student’s *t*-test for comparison between two groups, and one-way analysis of variance (ANOVA) followed by the *post hoc* Tukey test for multiple comparisons. *P* < 0.05 was considered statistical significance.

## Results

### The comparison of intestinal pathology between EgPSC-infected group and control group

**Figure 1:**
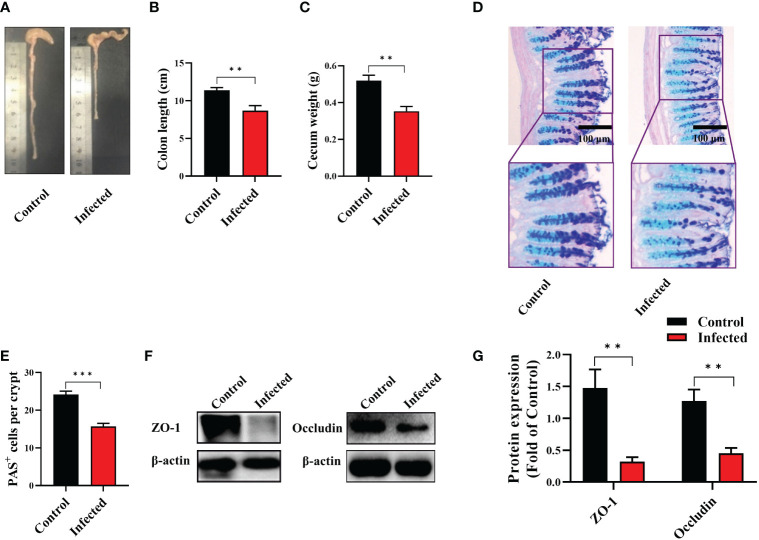
The protoscoleces of *Echinococcus granulosus* infection disrupted colonic epithelial barrier function in mice. The C57BL/6J mice were sacrificed 7 months post infection, colonic samples were collected for multiple analysis. **(A)** The representative images of colons. **(B)** The colon length (n = 6). **(C)** The cecum weight (n = 6). **(D)** Intestinal goblet cells and mucus were shown by PAS staining (n = 3, scale bar: 100 μm). **(E)** Number of PAS^+^ cells (goblet cells) per crypt. **(F, G)** Protein expression levels of ZO-1 and occludin in the colon (n = 4 - 6). The differences were analyzed using t-test. Values are presented as mean ± SEM. **P < 0.01, ***P < 0.001.

To detect the effect of EgPSC infection *via* non-oral transmission on intestinal pathological changes, C57BL/6J mice were intraperitoneally injected with 2,000 live EgPSC. The intestinal lesions were assessed after 7 months post-infection. We observed that EgPSC-infected group exhibited shorter colon length and decreased cecum wight, compared with the control group (*P* < 0.01, **Figures 1A–C**). Moreover, using PAS staining, we found that EgPSC infection decreased the amounts of PAS^+^ cells (globet cells) in the colonic villus of mice (*P* < 0.001, **Figures 1D, E**). Correspondingly, we also measured the expression of tight junction proteins, zonula occludens-1 (ZO-1), and occludin in the colon of mice. The EgPSC-infected group showed lower protein levels of ZO-1 and occludin than those in control group (*P* < 0.01, **Figures 1F, G**). The results suggest that EgPSC infection causes colonic injury in mice.

### Identification and validation of genes differentially expressed in the colon after EgPSC infection

**Figure 2:**
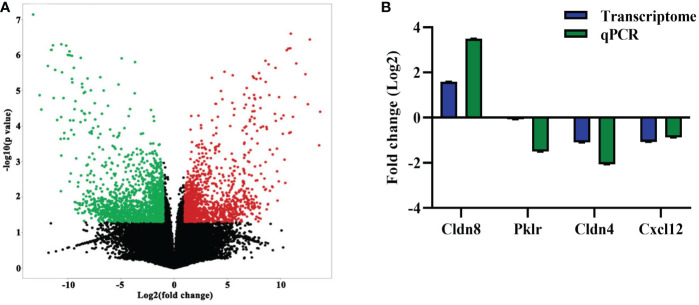
The identification and validation of differentially expressed mRNAs in the colonic tissues post the protoscoleces of *Echinococcus granulosus* infection. **(A)** The volcano plot shows the distributions of mRNAs between the Control and Infected group. The significantly upregulated and downregulated RNAs are presented as red and green dots, respectively, whereas the black dots represent the mRNA with no significant difference (*n* = 3). **(B)** qPCR validation of randomly selected mRNAs from the RNA-seq data (*n* = 4 - 6).

Following the observation of EgPSC-induced colonic disruption, we further characterized the transcriptomic profile in the colon of mice post-infection. The quality control was shown in **Supplementary Table S1**. Differentially expressed genes (DEGs) were classified significantly through parameters |fold change| > 2 and *P* < 0.05. As the volcano plots shown in **Figure 2A**, there were 3,019 genes differentially expressed, including 1,346 upregulated genes and 1,673 downregulated genes after EgPSC infection. Red dots represented DEGs significantly upregulated by EgPSC infection, whereas green dots indicated downregulated DEGs. Meanwhile, we randomly selected 4 DEGs (Cldn8, Pklr, Cldn4 and Cxcl12) for qPCR to confirm the results of transcriptome analysis. The similar expression trends of the genes were observed, as EgPSC infection upregulated the mRNA levels of Cldn8, and downregulated the mRNA levels of Pklr, Cldn4 and Cxcl12 (**Figure 2B**).

### KEGG pathway analysis of differentially expressed genes in the colon after EgPSC infection

**Figure 3:**
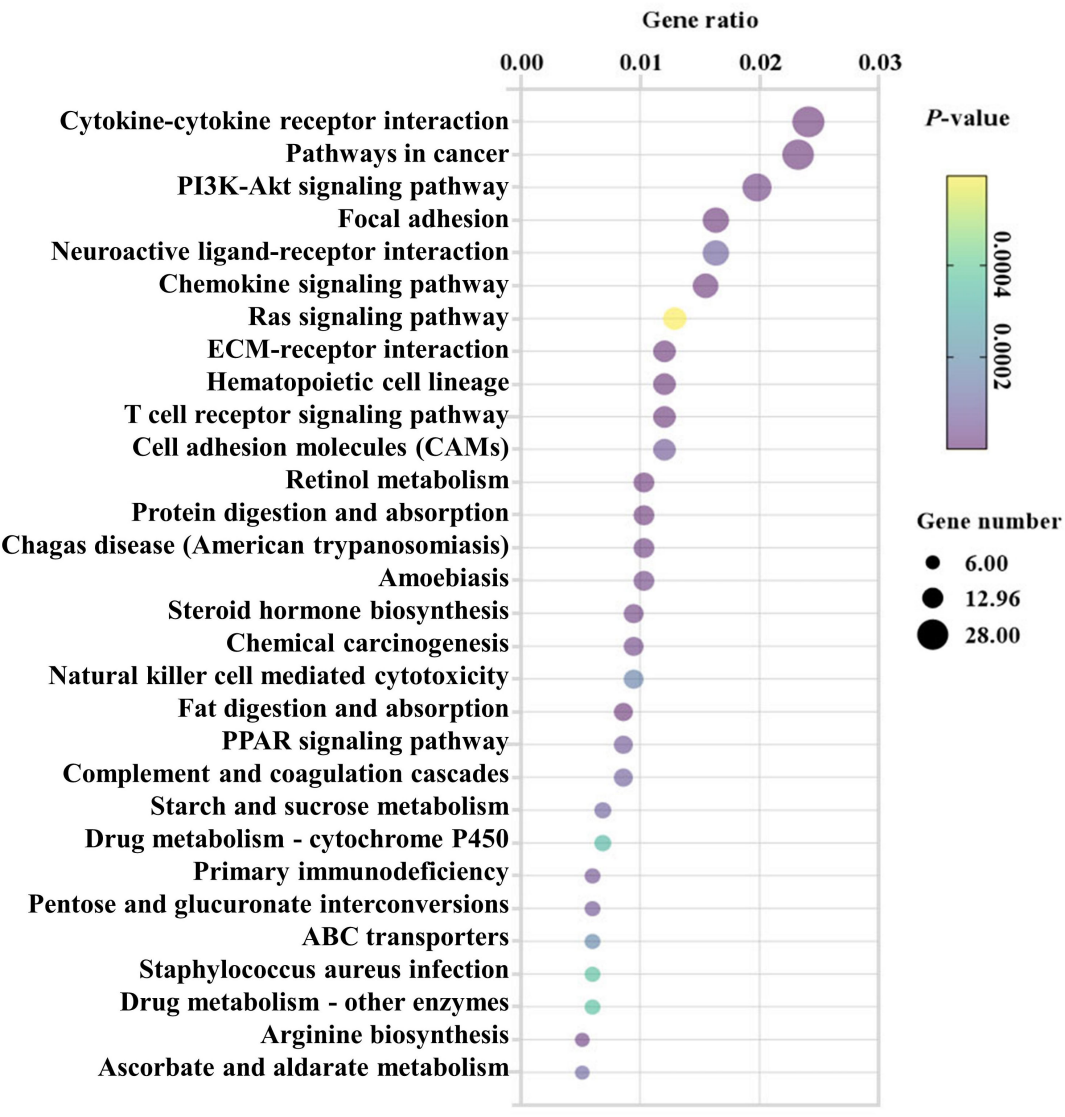
KEGG analysis (top 30) of the differentially expressed mRNAs in the colonic tissues post the protoscoleces of *Echinococcus granulosus* infection. Rich factor is the ratio of the differentially expressed gene number to the total gene number in a certain pathway. The color and size of the dots represent the range of the *P*-values and the number of DEGs mapped to the indicated pathways, respectively. The larger the point, the more genes involved in this pathway.

To predict the biological functions of DEGs, the pathways of DEGs were then analyzed by KEGG pathway enrichment analysis (20). The top 30 enriched pathways were shown in **Figure 3** and **Supplementary Table S2**. Of which, “Cytokine-cytokine receptor interaction (mmu04060)”, “Chemokine signaling pathway (mmu04062)”, “T cell receptor signaling pathway (mmu04660)”, “Cell adhesion molecules (CAMs) (mmu04514)”, “Natural killer cell mediated cytotoxicity (mmu04650)”, “Complement and coagulation cascades (mmu04610)” and “Primary immunodeficiency (mmu05340)” were considered as closely associated with intestinal inflammation and immune responses. In line with these, KEGG analysis exhibited for those genes also were found in intestinal infectious disease-associated pathways such as “Chagas disease (American trypanosomiasis) (mmu05142)”, “Amoebiasis (mmu05146)”, and “Staphylococcus aureus infection (mmu05150)”. Meanwhile, “Retinol metabolism (mmu00830)”, “Protein digestion and absorption (mmu04974)”, “PPAR signaling pathway (mmu03320)”, “Steroid hormone biosynthesis (mmu00140)”, “Fat digestion and absorption (mmu04975)”, “Starch and sucrose metabolism (mmu00500)”, “Drug metabolism-cytochrome P450 (mmu00982)”, “Drug metabolism-other enzymes (mmu00983)”, “Arginine biosynthesis (mmu00220)”, and “Ascorbate and aldarate metabolism (mmu00053)” involved in metabolism were significantly enriched post infection. Notably, in comparison with the control mice, “Focal adhesion (mmu04510)” associated with cell junction were significantly differed after EgPSC infection.

### Gene Ontology analysis of colonic differentially expressed genes after EgPSC infection

**Figure 4:**
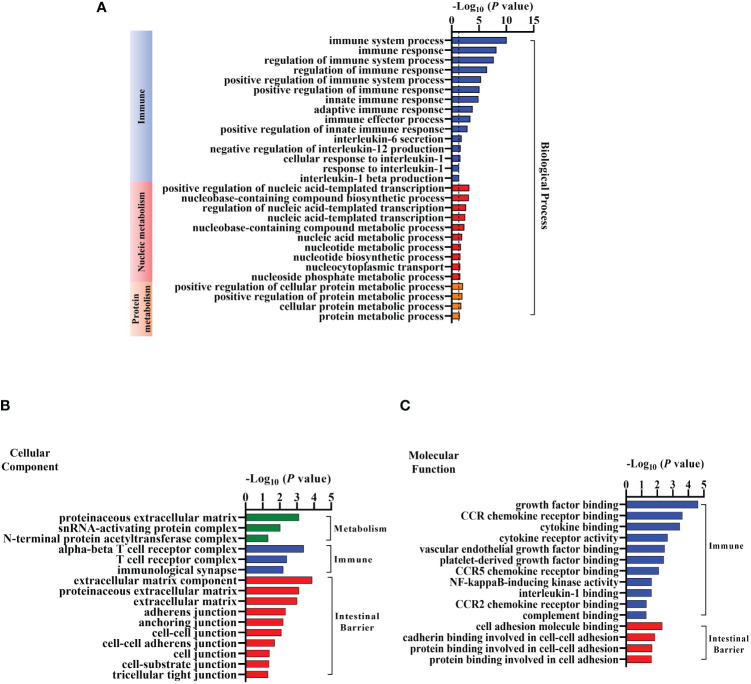
Gene Ontology (GO) Analysis of the differentially expressed mRNAs in the colonic tissues after the protoscoleces of *Echinococcus granulosus* infection. Go annotation of differently expressed mRNAs between the Control and Infected group (n = 3). **(A)** Biological process related to immune, nucleic or protein metabolism. **(B)** Cellular components connected with metabolism, immune and intestinal barrier. **(C)** Molecular function associated with immune and intestinal barrier.

We next conducted GO analysis of DEGs to evaluate gene enrichment in BP, CC, and MF. The top 30 enrichment scores of the three terms were shown in **Supplementary Figure 1**, respectively. There were many DEGs associated with metabolism, immune and intestinal barrier enriched in terms above (**Figure 4**). In detail, 15 terms related to immune, 10 terms associated with nucleic metabolism, and 4 terms linked to protein metabolism were identified in the BP terms (**Figure 4A**). Ten terms related to intestinal barrier, three terms associated with immune, and three terms linked with metabolism were observed in the CC terms (**Figure 4B**). Moreover, there were four terms related to intestinal barrier and eleven terms associated with immune found in the MF terms (**Figure 4C**). These results suggested that EgPSC infection induces reprogramming of intestinal barrier function and immunometabolic events in the colon of mice.

### Differentially expressed genes involved in tight junctions after EgPSC infection

**Figure 5:**
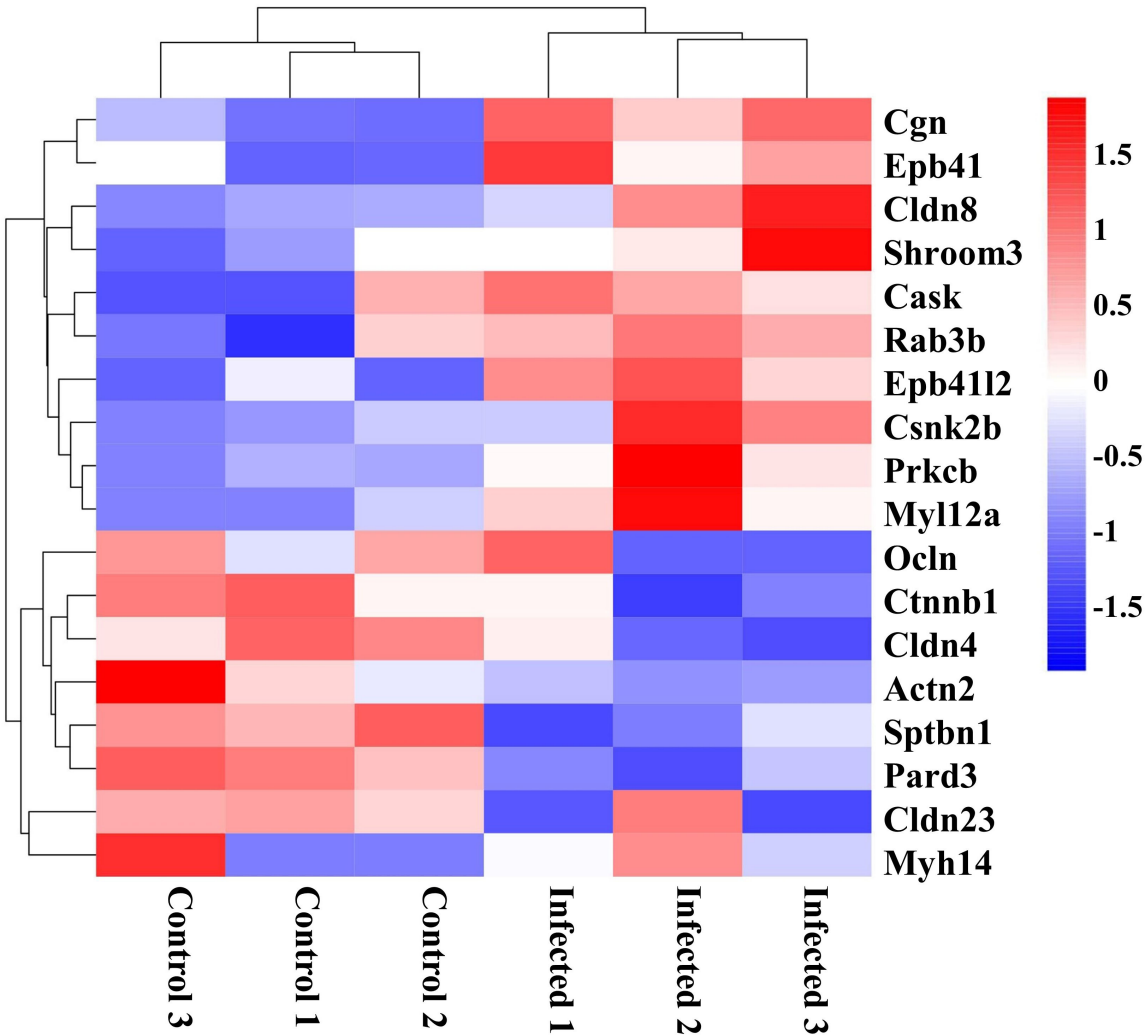
Clustering heatmap of differentially expressed genes related to tight junction organization in the colon post the protoscoleces of *Echinococcus granulosus* infection. Hierarchical clustering was performed as a heatmap, which shows distribution of the differential mRNA level between the Control and Infected groups. The color shows the degree to which the gene is expressed with range between -1.5 and 1.5. Red color means the upregulated mRNAs and blue color means the downregulated mRNAs.

Intestinal barrier plays a vital role in host defense against pathogen invasion, the disruption of tight junctions may cause the morphological changes of intestinal mucosa and the increase of intestinal permeability and pathogen invasion (30). Thus, DEGs related to tight junctions are analyzed in this study. As the clustering heatmap shown in **Figure 5**, EgPSC infection significantly decreased the expression of Ocln, Ctnnb1, Cldn4, Actn2, Sptbn1, Pard3, Cldn23, and Myh14. EgPSC infection also caused the upregulation of Cgn, Epb41, Cldn8, Shroom3, Cask, Rab3b, Epb41l2, Csnk2b, Prkcb, and MyI12a. Consistently with the results in **Figures 1D–G**, these findings indicated that EgPSC infection impairs the tight junction function of colon.

### Differentially expressed genes related to immune response after EgPSC infection

**Figure 6:**
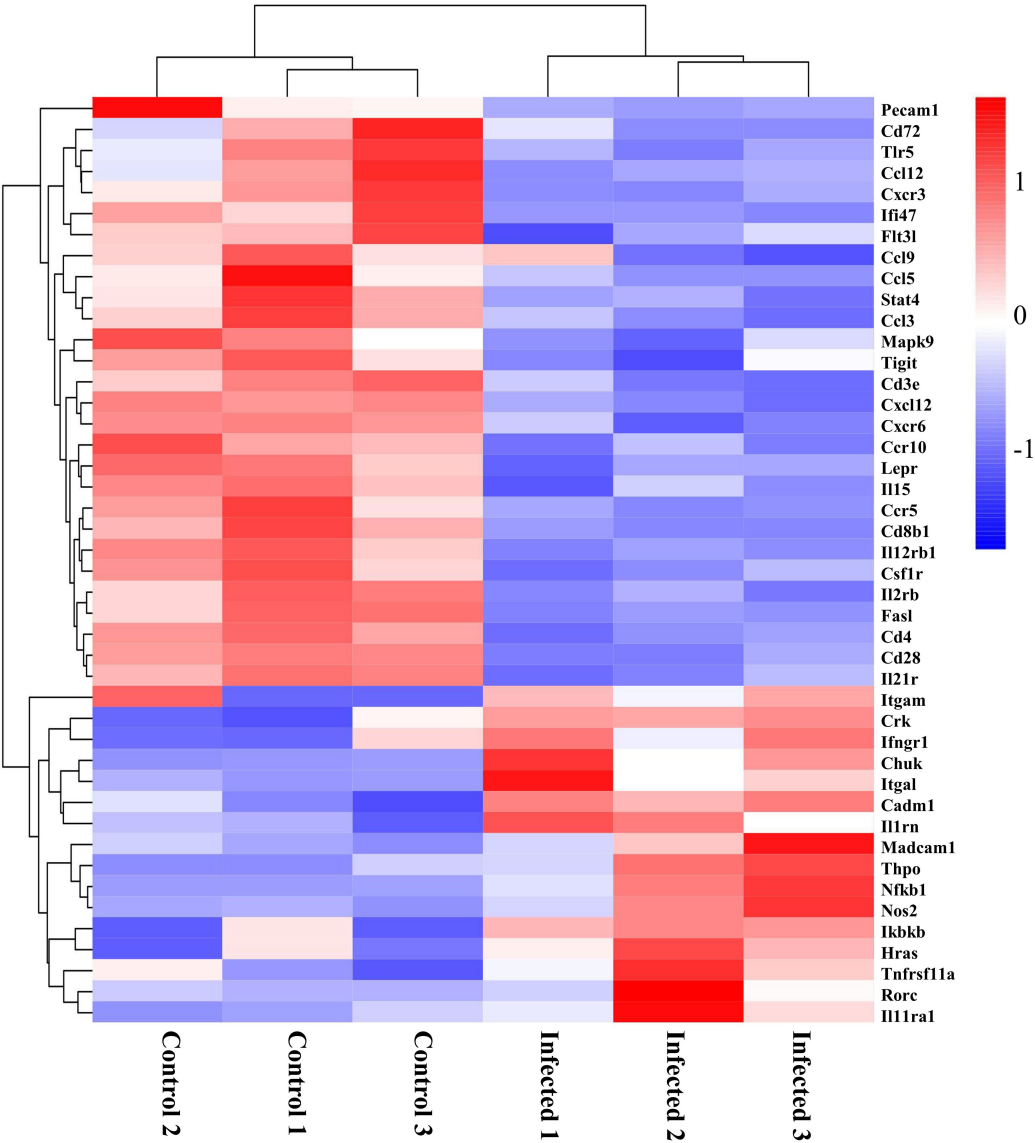
Clustering heatmap of differential genes associated with immunity in the colon post the protoscoleces of *Echinococcus granulosus* infection. Red color means the upregulated mRNAs, and blue color means the downregulated mRNAs.

The morphological changes of intestinal mucosa usually link with the activation of immune response (3). Following the observation of DEGs related to immune found in the GO analysis (**Figure 4**), we profiled these DEGs caused by EgPSC infection. As the clustering heatmap shown in **Figure 6**, EgPSC infection significantly downregulated the expression of innate immunity moleculars (Pecam1, Tlr5, Ccl12, Cxcr3, Ccl9, Ccl5, Ccl3, Cxcl12, Cxcr6, Ccr10, and Ccr5), adaptive immunity components (Pecam1, Cxcr3, Ifi47, Flt3l, Tigit, Cd3e, Lepr, Cd8b1, Csf1r, Fasl, Cd4, and Cd28), immunomodulatory factors (Cd72, Stat4, and Csf1r), immune response moleculars (Flt3l, Mapk9, and Cd3e), cytokines (Il15, Il12rb1, Il2rb, and Il21r). Correspondingly, EgPSC-infected group showed remarkably higher level of inflammatory-associated mRNA expression, such as Itgam, Crk, Ifngr1, Chuk, Itgal, Cadm1, Il1rn, Madcam1, Thpo, Nfkb1, Nos2, Ikbkb, Hras, Tnfrsf11a, Rorc, and Il11ra1. These results suggested that EgPSC infection alters intestinal immune response in mice.

### Differentially expressed genes involved in metabolism after EgPSC infection

**Figure 7:**
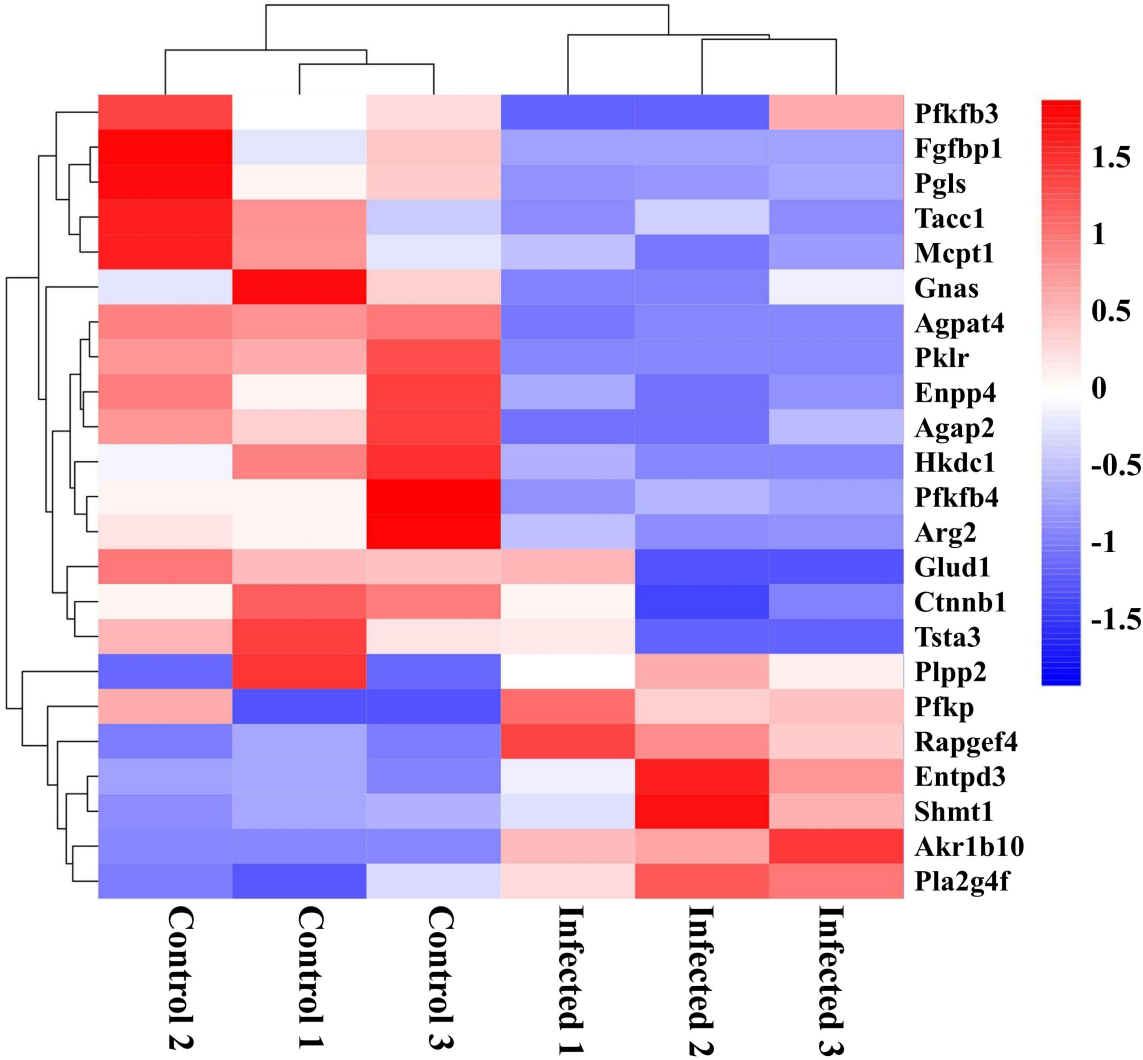
Clustering heatmap of differential metabolism-related mRNAs in the colon post the protoscoleces of *Echinococcus granulosus* infection. Red color means the upregulated mRNAs, and blue color means the downregulated mRNAs.

Recently, growing evidence demonstrates that EgPSC infection can regulate metabolism and influence metabolism-related diseases (22, 31). In this study, as shown in **Figure 7**, key genes associated with cancer cells metabolism (Pfkfb3, Fgfbp1, Gnas, Pfkfb4, Ctnnb1, and Tsta3), glucose metabolism (Pgls and Hkdc1), lipid synthesis (Agpat4, Pklr, and Ctnnb1), lipid hydrolysis (Enpp4), lipid transport (Agap2), fatty acid metabolism (Mcpt1), amino acid metabolism (Arg2 and Glud1), cell differentiation (Tacc1), and tissue repairment (Fgfbp1) were remarkably downregulated in EgPSC-infected mice. In addition, genes related to glycerolipid metabolism (Plpp2), glycolysis metabolism (Pfkp), adipocyte differentiation (Rapgef4), nucleotide metabolism (Entpd3), serine catabolism (Shmt1), retinol metabolism (Akr1b10), and lipid-grading metabolism (Pla2g4f) were upregulated after EgPSC infection. These results indicated that EgPSC infection induces intestinal metabolic reprogramming in mice.

### MBZ attenuated colonic injury induced by EgPSC in mice

**Figure 8:**
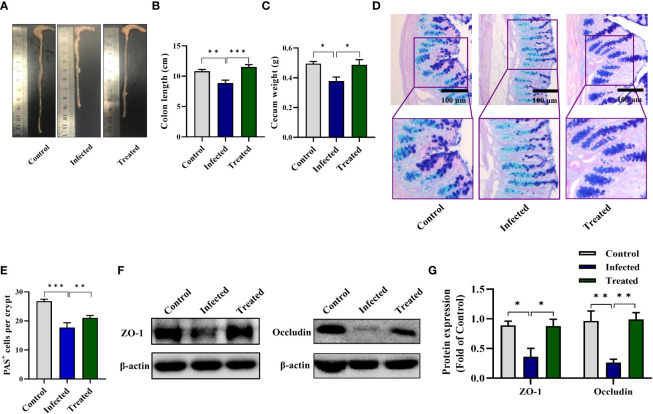
MBZ attenuated colonic histopathology in mice post the protoscoleces of *Echinococcus granulosus* infection. The protective effect of MBZ on colonic histopathology were evaluated by various parameters. **(A)** The representative images of colons. **(B)** The colon length (n = 6). **(C)** The cecum weight. **(D)** Intestinal goblet cells and mucus were shown by PAS staining (scale bar: 100 μm). **(E)** Number of PAS^+^ cells (goblet cells) per crypt. **(F, G)** Protein expression levels of ZO-1 and occludin in the colon (n = 4 - 6). *P < 0.05, **P < 0.01, ***P < 0.001.

**Figure 9:**
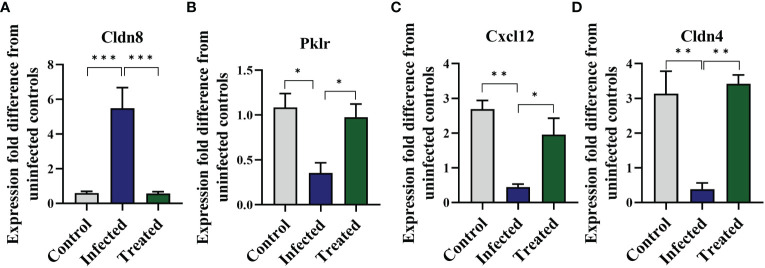
Mebendazole (MBZ) ameliorates abnormal gene expressions induced by the protoscoleces of *Echinococcus granulosus* infection in the colon of mice. qPCR was performed to verify indicated mRNA levels. **(A)** mRNA levels of Cldn8. **(B)** mRNA levels of Pklr. **(C)** mRNA levels of Cxcl12. **(D)** mRNA levels of Cldn4. *P < 0.05, **P < 0.01, ***P < 0.001.

MBZ is a priority drug for CE treatment. We next determined whether MBZ could prevent colonic impairment induced by EgPSC. We found that MBZ-treated group showed an increase of colon length and cecum weight, compared with the EgPSC-infected group (both *P* < 0.05, **Figures 8A–C**). Also, MBZ treatment significantly increased the number of PAS^+^ cells (globet cells) per crypt (**Figures 8D, E**). In line with these, the decrease of ZO-1 and occludin expressions induced by EgPSC were remarkably recovered by MBZ (both *P* < 0.05, **Figures 8F, G**). Besides, the abnormal expression of Cldn8, Pklr and Cldn4 found in transcriptomic analysis were attenuated by MBZ, although there was no statistically significant increase in Cxcl12 expression (**Figure 9**).

## Discussion

Using a non-orally transmitted *E. granulosus* infection model by intraperitoneally administrating EgPSC, the present study demonstrated for the first time that EgPSC infection *via* non-oral transmission impairs colonic integrity and induces related immunometabolism reprogramming. Interestingly, we unveiled that administration of MBZ, the choice drug for CE treatment (24), effectively ameliorates the colonic injury and immunometabolism reprogramming caused by the parasitic infection. Overall, these findings provide a novel insight for the function of intestinal tract in non-orally transmitted parasitic infection.

As a parasite infected *via* the fecal-oral route, *E. granulosus* infection usually impairs the gut of host (32). It’s reported that the orally-infected *E. granulosus* tightly installed in the intestine of dog, thereby establishing strong adhesion to crypts and impairing the intestinal mucus integrity (33). Additionally, the parasite can damage the epithelial tissue barrier, then penetrate and migrate to other organs in the Kazakh sheep (22). In the present study, we observed that intraperitoneally EgPSC infection reduces the colon length and cecum size of mice. The degeneration of globet-cell derived mucus indicates mucus invasion (4). Here, the EgPSC-infected mice exhibited less globet cells (PAS^+^ cells). Usually, tight junctions disruption is persistently observed in intestinal parasite infection models, since parasite-derived substances and parasite lysate could increase intestinal barrier permeability to facilitate parasite migration (34). A previous finding revealed that *Giardia*-infected mice exhibits sustained damage of Tight junctions (Tjs) in jejunum tissues (35). Here, we found that the EgPSC-infected group shows the decrease expression of ZO-1 and occludin. Besides, tight junctions-associated genes were mapped abnormal expression after EgPSC infection in transcriptomic analysis. For example, several classic tight junction related genes (Ocln and Cldn4) were significantly downregulated post-infection, whereas other tight junctions-relative genes (Cldn8, Epb41L2, Prkcb, and Shroom3) were upregulated. According to a previous study, Cldn8 over-expression leads to the downregulation of Cldn2 expression (36). The hyperexpression of Epb41L2, Prkcb, Shroom3 are implicated in adhesion damage (37–39). MBZ is a classic chemotherapeutic agent available for CE treatment (40). Notably, we found that MBZ treatment alleviates colonic epithelial barrier injury induced by EgPSC infection. These results indicated that, similar to orally infected model, non-orally transmitted infection of EgPSC also causes intestinal impairment, which can be reversed by MBZ administration.

Intestinal immunity is an important line of defense for host to expel the infection of oral transmitted parasites (41). Several studies have recognized the relationship between intestinal immune-related genes expression and CE (42). For example, Kazakh sheep with *E. granulosus* infection were found higher expression level of lectins receptors in intestines, facilitating the capability to respond and eliminate orally acquired *E. granulosus* (22). Here, we found that intraperitoneally EgPSC-infected mice show lower expression level of Stat4 and IL12rb1. It is reported that IL-12 is a key initiator of naïve CD4^+^ T cells differentiation into T helper 1 (Th1) cells, which depends on CD28 engagement (43). Th1 cytokines is suggested to induce protective immune process in *E. granulosus* infection (31). Also, there are decrease expression of Th1 chemokines (Ccl3, Ccl5, Ccl9 Ccl12, Ccr5, Cxcl12, Cxcr3, Cxcr6, and Cxcr10) and adaptive immunity components (Cxcr3, Ifi47, Fit3L, Tigit, Cd3e, Lepr, Cd8b1, Csf1r, Cd4, and Cd28) in the colon tissue of EgPSC-infected mice. In addition, stimulators such as pathogenic molecular can cause increased mRNA levels of NOS2 and enhancive NO production after the activation of Nfkb1 (44). The alternation Nfkb1 may stimulate immune cells and endothelial cells and induce overexpression of pro-inflammatory associated factors (Ifngr1, IL1rn, and Tnfrsf11a), resulting in systematic inflammation and intestinal lesion (9). In our study, some inflammatory genes were observed drastically increased in the colon of EgPSC-infected mice, such as Nfkb1, Nos2, Ifngr1, IL1rn, and Tnfrsf11a. Collectively, these results suggested that EgPSC infection *via* non-oral transmission can induce intestinal immune response.

Immunometabolism is a frontier field of research revealing the relationship between metabolic pathways and host immune, or even overall health (28). In the present study, KEGG and GO analysis forecasted that intraperitoneally EgPSC infection influences the expression of genes involved in “Metabolic pathways”, such as “Pathways in cancer”, “Fat digestion and absorption” and “Retinol metabolism”. Unlike other helminths’ carcinogenic impacts, *E. granulosus* infection was reported to serve as anticancer agent (45). Consistently, we found that several downregulated genes associated with cancer cells metabolism (Pfkfb3, Fgfbp1, Gnas, Pfkfb4, Ctnnb1, and Tsta3) in the colon of EgPSC-infected mice, indicating that intraperitoneally EgPSC infection may elicit protective effect in cancer development. In the past decade, immunometabolism has revealed that the differentiation and the function of immune cells are determined by metabolic pathways (28). As *E. granulosus* has restricted ability to synthesize lipids and needs to acquire essential lipids from its hosts (20). Our previous study revealed that EgPSC infection can enhance lipolysis in the adipose tissue of mice (20). Accordingly, upregulated lipolysis genes (Plpp2 and Pfkp) and downregulated genes of adipogenesis (Agpat4, Pklr, and Ctnnb1), lipid hydrolysis (Enpp4), lipid transport (Agap2), and fatty acid metabolism (Mcpt1) were identified in the colon of EgPSC-infected mice in the current study. It is documented that lipid molecules in the membrane of immune cells are essential for the activation or function of immune cells (46). Notably, the retinol may be important in host anti-parasitic effect, as CE-resistant sheep exhibited significant higher level of DEGs related to retinol metabolism (22). Our result revealed that non-orally transmitted *E. granulosus* has an effect on retinol metabolism in the colon tissue. Moreover, we found that numerous lipid metabolites are key events in the differentiation and functions of splenic CD19^+^ B cells in mice (47). Additionally, several studies have identified the significance of retinol in maintaining appropriate immune functions in hosts. Retinol-deprived cotton rats have showed higher susceptibility of filarial worms compared to cotton rats with sufficient retinal supplement. Retinol metabolism is crucially related to orally CE infection in Kazakh sheep (22). However, in the present study, we detected higher expression level of retinol metabolism gene (Akr1b10) in the colon of non-orally EgPSC-infected mice compared with the control mice. The different way of establishing infection models may contribute to the discrepancy. Moreover, MBZ administration reversed EgPSC-induced metabolic changes evidenced by restoring colonic Pklr expression. Overall, these results suggested that non-orally transmitted EgPSC infection induces colonic metabolic remodeling in mice. However, the exact role of these metabolic remodeling in intestinal function requires further study.

Similar to other intermediate hosts, *E. granulosus* forms the hydatid cysts filled with PSCs and fluids in the inner organs of human. Spontaneously or in a traumatic way, the cyst may rupture and the PSCs can establish the secondary infection over the body (31). This would cause serious consequence to human health. Herein, we highlighted the significance of complete intestinal functions in defensing the parasite. One limitation in this study might be that we only detected the colonic histopathology and functional gene expression profile post-chronic *E. granulosus* infection. To better comprehend host-parasite interplay during secondary *E. granulosus* infection, future works should focus on the dynamics of intestinal histopathology and functional gene expression profile at different time points after infection.

## Conclusions

In summary, this study employed the EgPSC-infected mouse model to reproduce the reinfection situation of *E. granulosus* by intraperitoneally injecting the PSCs of **E. granulosus*.* Notably, we reported, for the first time, that non-orally transmitted EgPSC infection caused colonic injury and immunometabolism reprogramming in mice, and MBZ administration alleviated above alternations, indicating that unblemished intestinal barrier function is of great importance in defensing secondary infection of **E. granulosus*.* These findings may provide a novel insight for treating the reinfection of the parasite.

## Data availability statement

The datasets presented in this study can be found in online repositories. The names of the repository/repositories and accession number(s) can be found in the article/**Supplementary Material**.

## Ethics statement

All the experimental protocols were performed in accordance with the recommendations of the Guide for the Care and Use of Laboratory Animals of the Ministry of Health (China), and approved by the Ethics Committee of Xuzhou Medical University (Xuzhou, China, SYXK (Su) 2020-0048).

## Author contributions

WP, XY, and HL: conceived and designed the experiments. YZ, TL, YYG, and DW: performed the experiments. YZ, DW, YXG, and TL: analyzed the data. YZ and DW: contributed reagents/materials/analysis tool. XY, WP, HL, ZG, LF, and FS: wrote the manuscript. All authors contributed to the article and approved the submitted version.

## Supplementary material

Supplementary Figure 1 Gene Ontology (GO) analysis of the differentially expressed mRNAs. Go annotation of differentially expressed mRNAs with top 30 enrichment numbers of **(A)** biological processes, **(B)** cellular components, **(C)** molecular functions. The GO terms with *P*-value ≤ 0.05 were considered significant.

## Glosarry

**Table:**

*E. granulosus*	*Echinococcus granulosus*
ZO-1	zonula occludens-1
KEGG	Kyoto Encyclopedia of Genes and Genomes
PSCs	protoscoleces
GO	Gene Ontology
MBZ	mebendazole
IEC	Intestinal epithelial cells
LPS	lipopolysaccharide
CE	cystic echinococcosis
ESPs	Excretory-secretory products
EgPSCs	larval *E. granulosus*
LAWEC	Laboratory Animal Welfare and Ethics Committee
PAS	Periodic Acid-Schiff stain
PCR	polymerase chain reaction
DESeq2	DESeq2 package for differential analysis of count data
FC	the fold change of expressions
BP	biological process
CC	cellular component
MF	molecular function
qPCR	quantitative reverse transcription-PCR
PBS	Phosphate Buffered Saline
PVDF	polyvinylidene difluoride
ANOVA	Analysis of variance
SEM	standard error of the mean
DEGs	differentially expressed genes
Tjs	Tight Junctions
NO	nitric oxide
Cldn23	Claudin-23
Sptbn1	Spectrin beta chain, non-erythrocytic 1
Ctnnb1	Catenin beta-1
Pard3	par-3 family cell polarity regulator
Cldn4	Claudin-4
Actn2	Alpha-actinin-2
Ocln	occludin
Cask	Peripheral plasma membrane protein CASK
Prkcb	protein kinase C, beta
Myl12a	myosin, light chain 12A, regulatory, non-sarcomeric
Csnk2b	Casein kinase II subunit beta
Cldn8	Claudin-8
Cgn	cingulin
Epb41l2	erythrocyte membrane protein band 4.1 like 2
Shroom3	shroom family member 3
Myh14	Myosin-14
Rab3b	RAB3B, member RAS oncogene family
Epb41	erythrocyte membrane protein band 4.1
Cd28	T-cell-specific surface glycoprotein CD28
Ccr5	C-C chemokine receptor type 5
Ccr10	C-C chemokine receptor type 10
Cxcl12	Stromal cell-derived factor 1
Ccl12	C-C motif chemokine 12
Ccl9	C-C motif chemokine 9
Ccl5	C-C motif chemokine 5
Cxcr3	C-X-C chemokine receptor type 3
Il12rb1	Interleukin-12 receptor subunit beta-1
Stat4	signal transducer and activator of transcription 4
Tlr5	toll-like receptor 5
Cd72	B-cell differentiation antigen CD72
Ccl3	C-C motif chemokine 3
Ifi47	interferon gamma inducible protein 47
Csf1r	Macrophage colony-stimulating factor 1 receptor
Mapk9	Mitogen-activated protein kinase 9
Cd3e	T-cell surface glycoprotein CD3 epsilon chain
Cd8b1	T-cell surface glycoprotein CD8 beta chain
Cd4	T-cell surface glycoprotein CD4
Pecam1	platelet/endothelial cell adhesion molecule 1
Tigit	T cell immunoreceptor with Ig and ITIM domains
Il21r	Interleukin-21 receptor
Cxcr6	C-X-C chemokine receptor type 6
Lepr	Leptin receptor
Il15	Interleukin-15
Il2rb	interleukin 2 receptor, beta chain
Flt3l	Fms-related tyrosine kinase 3 ligand
Thpo	thrombopoietin
Tnfrsf11a	Tumor necrosis factor receptor superfamily member 11A
Crk	Adapter molecule crk
Nfkb1	Nuclear factor NF-kappa-B p105 subunit Nuclear factor NF-kappa-B p50 subunit
Ikbkb	inhibitor of kappaB kinase beta
Chuk	conserved helix-loop-helix ubiquitous kinase
Rorc	Nuclear receptor ROR-gamma
Ifngr1	interferon gamma receptor 1
Itgal	Integrin alpha-L
Itgam	integrin alpha M
Il11ra1	Interleukin-11 receptor subunit alpha-1
Hras	GTPase HRas GTPase HRas, N-terminally processed
Cadm1	Cell adhesion molecule 1
Madcam1	mucosal vascular addressin cell adhesion molecule 1
Il1rn	Interleukin-1 receptor antagonist protein
Nos2	Nitric oxide synthase, inducible
Fasl	Tumor necrosis factor ligand superfamily member 6 Tumor necrosis factor ligand superfamily member 6
Pfkfb3	6-phosphofructo-2-kinase/fructose-2,6-biphosphatase 3
Pfkfb4	6-phosphofructo-2-kinase/fructose-2,6-biphosphatase 4
Pfkp	ATP-dependent 6-phosphofructokinase, platelet type
Fgfbp1	Fibroblast growth factor-binding protein 1
Ctnnb1	Catenin beta-1
Akr1b10	aldo-keto reductase family 1, member B10
Tsta3	tissue specific transplantation antigen P35B
Gnas	GNAS (guanine nucleotide binding protein, alpha stimulating) complex locus
Pla2g4f	Cytosolic phospholipase A2 zeta
Rapgef4	Rap guanine nucleotide exchange factor (GEF) 4
Agpat4	1-acyl-sn-glycerol-3-phosphate acyltransferase delta
Plpp2	phospholipid phosphatase 2
Glud1	Glutamate dehydrogenase 1
Arg2	Arginase-2, mitochondrial
Enpp4	Mus musculus ectonucleotide pyrophosphatase/phosphodiesterase 4
Entpd3	ectonucleoside triphosphate diphosphohydrolase 3
Pgls	6-phosphogluconolactonase
Pklr	pyruvate kinase L/R
Shmt1	serine hydroxymethyltransferase 1 (soluble)
Hkdc1	Putative hexokinase HKDC1
Tacc1	Transforming acidic coiled-coil-containing protein 1
Mcpt1	mast cell protease 1
Agap2	ArfGAP with GTPase domain, ankyrin repeat and PH domain 2
SRA	Sequence Read Archive.
