# Supplementary figures and images for: The non-oral infection of larval Echinococcus granulosus induces immune and metabolic reprogramming in the colon of mice
Source: Front Immunol. 2023 Jan 13;13:1084203. doi: 10.3389/fimmu.2022.1084203 (PMC9880436; doi:10.3389/fimmu.2022.1084203)

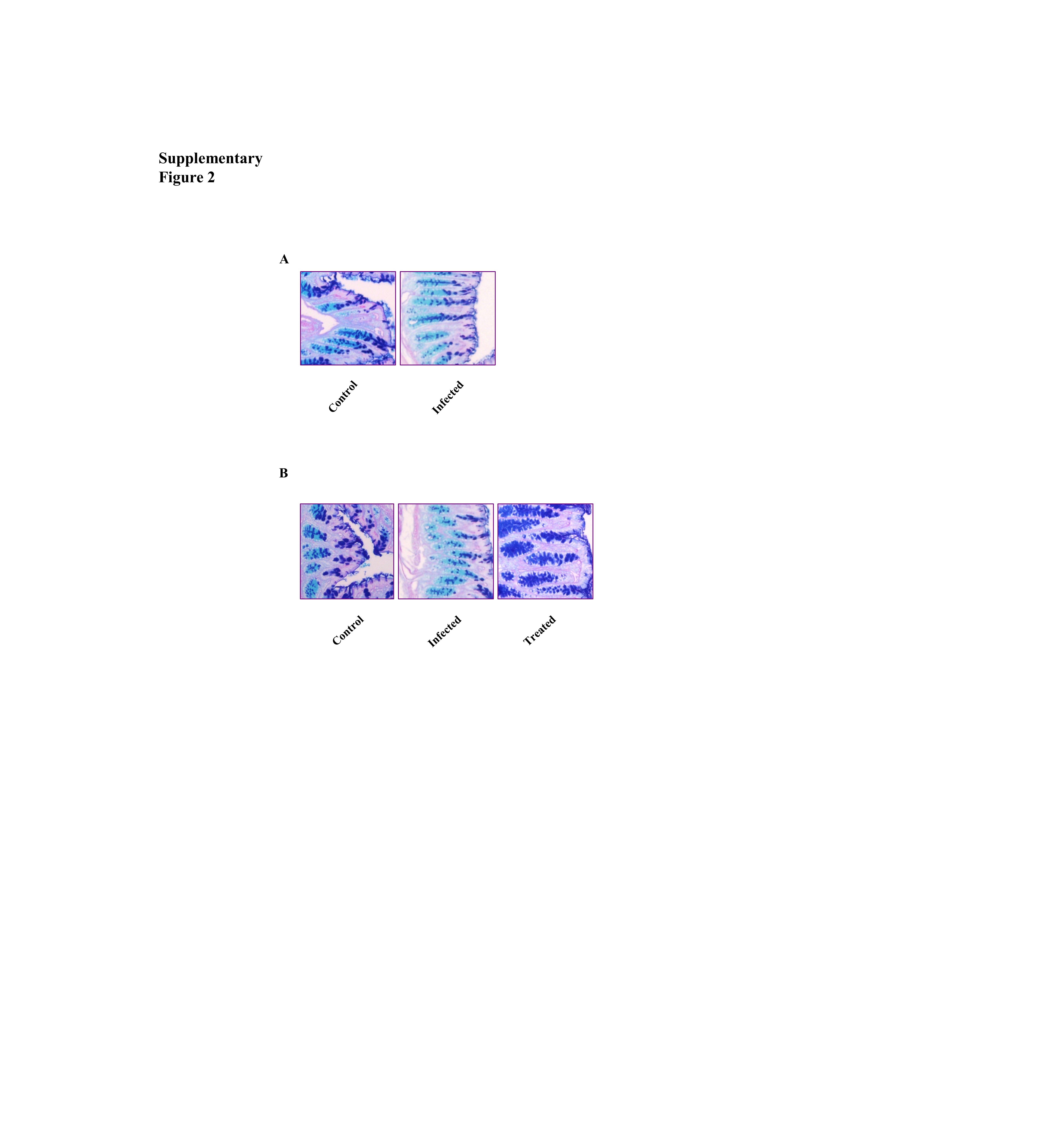

Supplement: Supplementary file 1 [file Image_2.jpeg]

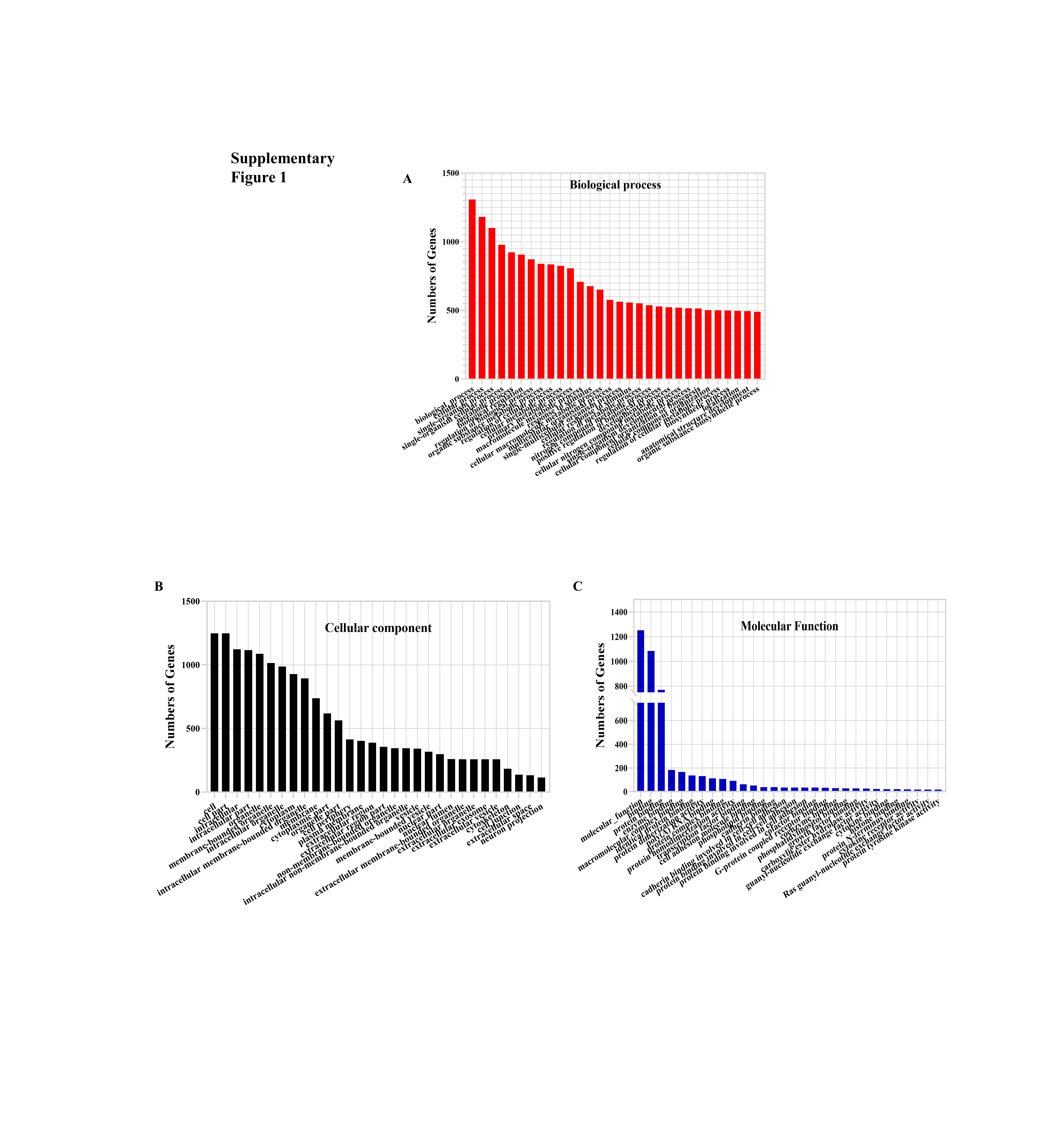

Supplement: Supplementary Figure 1 — Gene Ontology (GO) analysis of the differentially expressed mRNAs. Go annotation of differentially expressed mRNAs with top 30 enrichment numbers of (A) biological processes, (B) cellular components, (C) molecular functions. The GO terms with P-value ≤ 0.05 were considered significant. [file Image_1.jpeg]
